# Long-term practice of intuitive inquiry meditation modulates EEG dynamics during self-schema processing

**DOI:** 10.1016/j.heliyon.2023.e20075

**Published:** 2023-09-12

**Authors:** Junling Gao, Hang Kin Leung, Bonnie Wai Yan Wu, Jenny Hung, Chunqi Chang, Hin Hung Sik

**Affiliations:** a Centre of Buddhist Studies, The University of Hong Kong, Hong Kong; b Division of Humanities, The Hong Kong University of Science and Technology, Hong Kong; c School of Biomedical Engineering, Shenzhen University, Shenzhen, China

**Keywords:** Event-related potential (ERP), Intuitive inquiry meditation (Zen Chan), Self-schema, Doubt, Belief, EEG spectral dynamics

## Abstract

**Objective:**

Intuitive inquiry meditation is a unique form of Buddhist Zen/Chan practice in which individuals actively and intuitively utilize the cognitive functions to cultivate doubt and explore the concept of the self. This event-related potential (ERP) study aimed to investigate the neural correlates by which long-term practice of intuitive inquiry meditation induces flexibility in self-schema processing, highlighting the role of doubt and belief processes in this exploration.

**Methods:**

Twenty experienced and eighteen beginner meditators in intuitive inquiry meditation were recruited for this ERP study. The interactions of doubt and belief processes with concepts of the self and Buddha were investigated. A 128-channel electroencephalography (EEG) system was used to collect EEG data. The ERP data were processed and analyzed using EEGLAB.

**Results:**

The data showed a double dissociation between beginners and experienced meditators (monks) in the concepts of the self and Buddha: intuitive inquiry meditation reduced the brain activity of beginners when viewing Buddha image but not when viewing a picture of themselves. However, in experienced meditators, intuitive inquiry meditation reduced brain activity when they viewed images of themselves but not when they viewed Buddha image. Further event-related spectral perturbation (ERSP) analysis revealed that experienced meditators had a greater theta spectral power and higher intertrial coherence (ITC), indicating that they could more flexibly modulate ongoing cognitive processes than beginner meditators.

**Conclusion:**

Intuitive inquiry meditation could help beginner meditators detach from the concept of Buddha but not from that of the self. However, in experienced meditators, the opposite was true. ERSP analysis showed that only experienced meditators exhibited significant alterations in brain activity dynamics during intuitive inquiry meditation, which might enable these practitioners to become spontaneously detached from the concept of the self. These findings revealed the neural mechanism by which long-term practice of intuitive inquiry meditation can influence the doubting process and its effect on self-schema processing.

## 1 Introduction

Meditation is increasingly recognized for its potential to reduce stress, regulate emotions, and promote mental clarity in contemporary society, where individuals often grapple with cognitive dissonance and uncertainty. Currently, individuals face fewer physiological challenges, such as hunger and intensive physical labor, but frequently encounter psychological challenges (ambiguity and disinformation) that induce stress, uncertainty, and disinformation. This is especially true in recent years, where recent events have challenged past expectations and beliefs, instigating doubts and cognitive dissonance. Fortunately, the prefrontal lobe in the human brain can help individuals cope with contradictions and feelings of skepticism, enabling us to make careful decisions [1,2]. From a cognitive perspective, doubt and belief are two essential components of human cognition. We have beliefs and schemas, which are formed from our experiences and allow us to make swift decisions. At the same time, doubting process is needed to continuously update past beliefs so that they more precisely reflect reality. The doubting process, instead of dogmatic attachment/belief, is essential for critical thinking and the development of insight, which is the hallmark of human intelligence. In this sense, doubting process is as vital as beliefs for maintaining cognitive balance.

Attachment (a form of belief) appears in the very early development of humans and animals and helps individuals learn from their parents, thus improving their chances of survival. Belief is a broadly used concept and supports intuitive understanding [[3], [4], [5]]. Broadly speaking, a variety of mental representations, including knowledge, rules, opinions, and ideas, can be regarded as beliefs [6]. These beliefs or cognitive representations can be retrieved and used as schemas in the brain, similar to tools in a warehouse, to facilitate our understanding and processing of current information, as suggested by traditional theories on cognitive representation. Thus, belief is an autonomic state that facilitates understanding and responses to a continuous flow of new information. These automatic processes can lead to a rapid automatic response if not blocked by the counterbalancing processes of doubt and dismissal.

According to false tagging theory [4], belief is a primary, inevitable, and fast-to-follow cognitive process intertwined with comprehension. It is sustained by representations in the post-Rolandic cortex and is used for further cognitive manipulation and execution. Unfortunately, this process is susceptible to be exploited by disinformation like scam phone calls, affecting individuals who are most vulnerable, such as senior citizens nowadays [7]. Previous studies have found that doubt and disbelief are mediated by the prefrontal lobe, which may deteriorate in old age [5]. In contrast, doubt is a more retroactive process through which a person checks and rejects potentially false information that could cause misunderstandings, fraud and deception.

Doubt, disbelief and critical analysis are more effortful processes that could result in uncertainty and confrontation, when compared with belief process. However, given the overwhelming amount of propaganda and advertisements in the modern world, doubt and analytical thinking may be important to a person's ability to live a healthy life because they help individuals to probe the real situation, steering them away from dangerous belief in disinformation and erroneous dogmatic assumptions.

The doubting process plays an essential role in critical thinking and helps us detach from dogmatic routines and assumptions, which prevent us from being creative. Creativity requires divergent thinking and sometimes involves disruption to the traditional, routine ways of thinking and acting. In contrast, the dogmatic need for structure impedes creativity following schema-inconsistent information [8]. There are several ways to train one's doubt and critical thinking processes [9,10]. Interestingly, intuitive inquiry meditation, a form of Chinese Buddhist practice, utilizes the doubting process to explore fundamental questions, encouraging practitioners to engage all cognitive resources in this inquiry, ‘inquiring the beginning of the word’. In this approach, practitioners identify a fundamental issue and question it continuously. The practitioner has to use his or her full attentional or cognitive resources to fully inquire about the fundamental question selected, and the practitioner must keep inquiring and doubting his or her beliefs while ignoring anything else unrelated to the subject of the question. To break away from the restriction of dogmatic schema, intuitive inquiry meditation emphasizes mental flexibility, observing the idle mind while contemplating the ever-changing mind and the impermanence of reality [11,12].

Among the various kinds of meditation, intuitive inquiry meditation is unique. Practitioners of intuitive inquiry meditation are encouraged to examine and doubt all concepts and ideations without using conceptual analysis but rather directly and intuitively approach the object of meditation with doubt and inquiry. Even concepts of Buddha and self should be questioned, doubted and released before real enlightenment can be achieved. Intuitive inquiry meditators are not only guided to inquire and doubt the object of meditation; for example, when considering Buddha, inquiry and doubt should also be directed toward the one who is meditating, the self. Chan masters often point out that a small amount of doubt leads to a low level of enlightenment, while a large amount of doubt leads to a high level of enlightenment [13,14].

From a neuroscientific perspective, belief and doubt are two fundamental cognitive forces that shape our thoughts and subsequent behaviors. During infant development, belief is formed earlier than doubt. This is true not only in human development but also in animals to facilitate survival. Doubt appears much later in development, with human brain maturation, and the prefrontal cortex plays an important role in the development of the doubting process [4]. According to false tagging theory, doubt is an important process that helps an individual prevent interference from false or unnecessary information during the decision-making process or while concentrating on an ongoing task [4].

In the current study, we analyzed the most popular form of Chinese Chan (Zen) meditation practice, Can-Hua-Tou, ‘Who is chanting the name of Buddha?‘. This is a priming question that helps the meditator to develop a doubting and inquisitive mind toward the practice of repetitive religious chanting of the name Amitabha Buddha, the most popular religious practice in Mahayana Buddhist traditions. Our previous study investigated the effect of religious chanting by practitioners who strongly believed in Amitabha Buddha. A previous event-related potential (ERP) study showed that religious chanting could mitigate negative responses to threatening events, probably by creating a positive schema during repetitive religious chanting. In contrast, repetitive chanting of the name of Santa Claus did not have a similar effect, although the participants also associated Santa Claus with a happy and familiar feeling. The difference might stem from the lack of belief of these practitioners in Santa Claus [15].

Further study revealed that religious chanting may reduce the eigenvector centrality of the posterior cingulate cortex (PCC), a region known to support self-referential processing [15]. It is suggested that the neural mechanism of religious chanting involves concepts of the self and belief. Indeed, some religious practices, such as those in the Pure Land sect of Mahayana Buddhism, emphasize the importance of belief. According to the Pure Land sect, having faith and belief in Amitabha Buddha is a prerequisite to the practice of religious chanting and for the practitioner, ‘the self’, to be reborn in the Pure Land.

Intuitive inquiry meditation, on the other hand, drawing on the popularity of Pure Land Buddhism, guides the practitioner to directly inquire into the identity of ‘who is chanting the name of Buddha’. If it is the practitioner, ‘the self’, who is chanting the name of Buddha, this priming question will prompt the meditator to ask ‘who am I’, ‘what is the self’, etc. Basically, the meditator must develop a meta-awareness to doubt and ask who is chanting the name of Buddha and who/what is consciously experiencing everything that is happening. At a certain point, the practitioner and the one who is doubting become the target object of inquiry during deep reflexive meditation. Other meditation methods, such as those in Tibetan Buddhism (Mahamudra), also involve inquiry [16], but intuitive inquiry meditation solely involves the meditator intuitively engaging in a process of inquiry and doubt without using conceptual analysis. An individual with long-term practice of intuitive inquiry meditation may thus train his or her ability to concentrate on intuitive inquiry, that is, reflecting without conceptual analysis. Intuitive inquiry meditation also helps the practitioner cultivate a flexible and detached mental state while remaining doubtful and skeptical toward what is perceived and believed thereafter. These assumptions have widely been affirmed, but no neuroscientific study has yet attempted to examine them.

We focused on the popular Chinese Chan (Zen) meditation practice, Can-Hua-Tou, ‘Who is chanting the name of Buddha?’. This question primes the meditator to develop a doubting and inquisitive mind, which is essential for the practice of repetitive religious chanting, a widespread religious practice in Mahayana Buddhist traditions. We explored how this practice affects both belief and doubt processes and its potential influence on brain regions related to self-processing. Moreover, as intuitive inquiry meditation promotes cognitive flexibility, we aimed to investigate whether experienced intuitive inquiry meditators exhibit more flexibility when engaging and disengaging from activity in various brain regions due to long practice. This investigation contributes to our understanding of the impacts of meditation on human cognition and the associated neural underpinnings.

Using the ERP technique, which can measure dynamic brain activity, this study aimed to investigate how intuitive inquiry meditation leads to doubt and deconstruction of the concept of the self and influences brain regions related to self-processing. The concept of the self is of interest to Buddhism as well as clinical psychology. We investigated both doubt and belief processes, two counterbalancing but fundamental functions of human cognition. In addition, as intuitive inquiry meditation training enhances mental flexibility, another aim of this study was to determine whether experienced intuitive inquiry meditators could more effectively modulate brain activity as a result of their long practice.

## 2 Materials and methods

The experiment was approved by the university ethics committee. Following our pilot study, 20 experienced intuitive inquiry meditators (monks) and 18 beginners in the practice (layman Buddhists), all of whom were male, were recruited at a retreat. The monks were 48 (*SD* = 13) years old on average, while the laymen were 47 (*SD* = 11) years old on average. The monks all had 5–28 years of practice with intuitive inquiry meditation, mainly in the Chinese Chan tradition. Self-inquiry meditation is also called ‘Can-Hua-Tou’; in both, the practitioner thoroughly and deeply questions the true identity of “Who is chanting the name of Buddha?“. This form of meditation typically requires decades of practice before enlightenment is achieved. The layman participants in the control group had some experience in inquiry meditation, but all were at the beginner level (less than half a year of practice). Participants with mental disorders or neurological diseases were excluded from this study.

ERPs were measured to identify potential neural correlates of the meditators’ reactions to the concepts of “the self” and “Buddha”; this approach was taken because the concepts of “the self” and “Buddha” both have religious significance but should have different neurological implications, as one involves the identity of the self (internal) as opposed to Buddha (an external object). This experiment was conducted with a 2 × 2 (within) × 2 (between) factorial design: two meditation conditions (*inquiry* vs. *normal*), two types of pictures (*Buddha* vs. *the self*), and two groups of participants (*experienced monk* vs. *layman control*). The two meditation conditions included an inquiry meditation condition and a normal control condition. A priming phrase (“Who is chanting the name of Buddha?“; 念佛是誰in Traditional Chinese) was used to elicit an inquisitive meditative state in the inquiry meditation condition, whereas the normal condition used the phrase “Jia Yi Bing Ding” (甲乙丙丁 in Traditional Chinese; a commonly used sequence similar to “one, two, three, four”). Thus, the four Chinese characters presented were comparable in the two conditions. Two types of pictures were shown after the priming phrase, a picture of Buddha and a picture of “the self”, that is, a picture of the meditator, which was taken before the experiment and transformed by software (PhotoLab™) to ensure that the resolution and pose of the two pictures were comparable.

The experiment had a block design based on the real-world situations experienced during the practice of intuitive inquiry meditation, as meditators do not shift quickly from an inquiry meditation state back to the normal mental state in seconds. Given the limited time of ERP experiments, a unique paradigm was designed using a prime to imprint the inquiry/Chan state over other information processing states. Each block lasted 150 s and contained 30 trials. Each of the four unique scenarios consisted of two blocks, resulting in the random presentation of 8 blocks. In each trial, the priming phrase was presented for 1.7–2.3 s, then an image (of the participant or Buddha) was presented for 2.0 s, and then a fixation cross (’+‘) was presented for 0.5–1.5 s; the time windows included jitter. The participants performed a short practice run before starting the experiment. The ERP experiment lasted approximately 24 min with a 20-s rest period between blocks. The main purpose of the rest period was to continuously remind the participant engage in inquiry meditation while viewing and pondering the concepts of the self or Buddha. In the inquiry condition, this thought process involved following the daily practice of deeply questioning/doubting the true identity of ‘Who is chanting the name of Buddha’. This repeated questioning generated doubting process and induced inner investigation. This thought process was measured by subjectively asking participants how well they did during the experiment and, more importantly, by objectively exploring the correlations between ERP data and time/frequency analysis. The monks had chosen inquiry (Chan) meditation for their main daily practice, while the control group was participating in an inquiry (Chan) meditation camp; thus, both groups fully understood the instructions in this experiment. However, given the experimental restrictions (i.e., time), both groups verbally reported that they only achieved half of the inquiry (Chan) meditation state during this experiment. The participants also performed a short practice run before starting the experiment.

The experiment took place in a quiet room at a temple for monks, who were attending an intuitive inquiry meditation retreat. Although the laymen were recruited at the same retreat, they participated in the experiment in a quiet room at an EEG laboratory. Data from 3 of the monks could not be used due to equipment issues at the temple. The same equipment was used for both groups. E-prime (Psychology Software Tools, Inc., USA) was used to present stimuli on a Windows PC. The participants were seated approximately 50 cm away from the monitor and could fully view the images without any eye movement. EEG data were acquired using a 128-channel EGI EEG system (Electrical Geodesics, Inc., USA), and the impedance of electrodes was mostly kept under 30 kΩ, as recommended by the manufacturer. The EEG data sampling rate was set at 1000 Hz, and the reference was set at the Cz electrode using the international 10–20 electrode placement system.

The EEG data were processed and analyzed using EEGLAB based on the MATLAB platform. Data were preprocessed before analysis. The raw EEG data were converted into a format that could be read by EEGLAB and resampled from 1000 Hz–250 Hz, and a bandpass filter of 1–30 Hz was applied. Data were cleaned manually to remove muscle artifacts and line noise. Channels with consistent artifacts were reconstructed using spherical interpolation. Further data cleaning was performed using independent component (IC) analysis. The ICs representing eye movement, muscle movement, and channel artifacts were discarded before data reconstruction, which used the remaining ICs. Finally, EEG epochs that were time-locked to visual stimulus onset (−300 ms–900 ms) were extracted and averaged to obtain the ERP data, and baseline correction was applied based on the interval of −300 ms–0 ms. The average reference value was applied to each dataset afterward. The effects of inquiry meditation on ERP components were obtained by subtracting the ERP data obtained in the normal condition from those obtained in the inquiry condition. Independent-sample t tests were used to examine significant group differences.

The time-frequency analysis of the ERP data was performed based on event-related spectral perturbation (ERSP) and intertrial coherence (ITC). EEGLAB calculates ERSP by computing the power spectrum in each ERP trial and then averaging them across trials. Following EEGLAB's recommendation, a frequency varying wavelet cycle was used to obtain ERSP [17]. We used a 1-cycle wavelet with the scale expansion factor set to 0.8. This resulted in the evaluation of frequencies in the range of 3–27 Hz, with 1 cycle at the lowest frequency to 1.8 cycles at the highest, and generated 200 time points between −114 and 710 ms. To visualize a wider range of variation, these measurements were log-transformed [18].

ITC is a frequency-domain index of the degree of synchronization of neural responses to a set of experimental events, with 0 indicating no synchronization and 1 indicating perfect synchronization. ITC values are phrase-locked, and a particular latency and frequency are specified to calculate ITC [19]. Again, we used a 1-cycle wavelet and a 0.8 scale expansion factor to compute ITC. Repeated-measure analysis of variance (ANOVA) with false discovery rate (FDR) correction was applied to detect significant differences between groups and conditions. This analysis was performed after significant ERP results were obtained in landmark channels of the international 10–20 system.

In addition to channel-based analyses, source localization analyses were conducted based on dipole fitting and *k*-means clustering. Following the methods in EEGLAB, the dipole source algorithm was applied to locate brain activity during the inquiry condition compared to during the normal condition, specifically focusing on the frontal region, which is associated with the doubting process (see supplementary sFigs. 16 and 17).

a. Statistical Methods

This study employed a multifactor design with two conditions (doubt vs. belief), two types of pictures (Buddha vs. the self), and two participant groups (monks vs. laymen). Further analysis was conducted on different ERP components: the P100 (70–140 ms), N170 (140–220 ms), and P300 (220–400 ms). The primary focus was on the effects of the inquiry condition, which involved a doubting process, on ERP components recorded when viewing images of Buddha and the self. These effects were then compared between the monks and laymen.

Traditional ERP analysis was utilized to generate 2D topographic maps for different ERP components. A specific landmark channel was chosen for further time-frequency analysis of the potential differences in neurodynamics between conditions and groups. This time-frequency analysis of the ERP data was performed based on ERSP and ITC values. ERSP data were calculated by computing the power spectrum in each ERP trial and then averaging them across trials. ITC, a frequency-domain measure of the degree of synchronization of neural responses to a set of experimental events, was calculated using a 1-cycle wavelet at lower frequencies. Additionally, dipole source localization was applied to calculate neurodynamics in the frontal area, which is related to the doubting process, and to compare the results between groups and conditions.

The statistical analysis of ERP data included using paired t tests for within-group comparisons and independent-sample t tests for between-group comparisons after subtracting values in the normal condition from those in the inquiry condition. A repeated-measure ANOVA with FDR correction was applied to detect significant differences between groups and conditions in the time-frequency analysis. This analysis was performed after significant ERP results were obtained in the landmark channel Fz, which is a representative location over the frontal/prefrontal cortex, a brain region known to be involved in higher cognitive processes such as decision-making and attention. ANOVA with FDR correction was also used to analyze ITC and ERSP data from a cluster of ICs of the frontal area and to explore potential interactions.

## 3 Results

a. ERP components and time windows

**Fig. 1:**
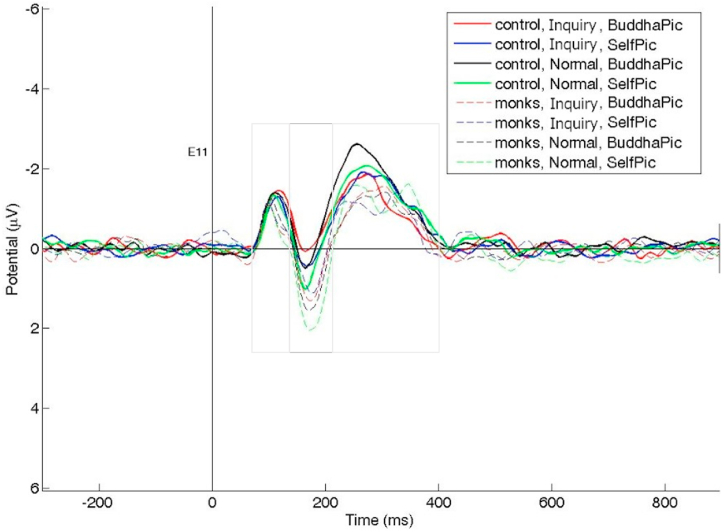
ERP waveforms in the two meditation conditions (inquiry vs. normal) for the two picture types (Buddha vs. the self) in the two groups (experienced monk vs. layman control) at Fz. Thicker solid lines represent the control group.

The ERP results differed according to the meditation condition and type of image viewed mainly in early time windows (140–220 ms and 220–400 ms). These time windows were chosen based on our ERP study on the priming effect of compassion and wisdom meditation [20,21]. The 70–140 ms (P100) and 140–220 ms (N170) windows represent ERP components during earlier stages of visual information processing, while the 220–400 ms window (P300) reflects an ERP component commonly used to evaluate later stages of information processing [22,23]. ERP analysis involved comparisons of the effect of inquiry meditation on EEG responses when viewing images of Buddha and the self in both the layman control group and the experienced monk group. The control group generally had a more negative trend in ERP data in channel Fz (medial frontal site). The two groups had different neural responses to the two types of images: participants in the control group had weaker responses to viewing the image of Buddha, while participants in the monk group had weaker response to viewing the image of themselves (see Fig. 1 and Supplementary Figs. S1 and S2). The significance of the channel-based difference was determined by comparing the average amplitude of different time windows.

### 3.1 Self-inquiry meditation and its effects

**Fig. 2:**
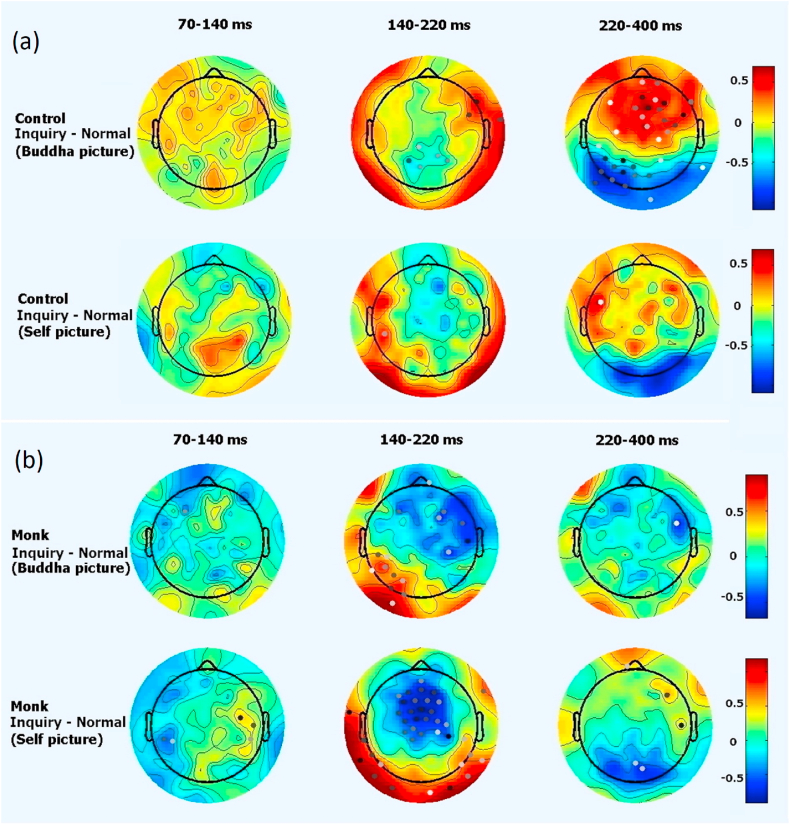
EEG responses in the two conditions. There was a clear double dissociation between the two groups: both between the inquiry and normal conditions and between two types of pictures (the self vs. Buddha). The power difference is represented by colors: red represents positive values and blue represents negative values. Dots indicate channels with significant differences (*p* < 0.05), and darker dots indicate smaller p values, (a): layman control group, (b): experienced monk group.

Interestingly, as shown in Fig. 2, the effect of intuitive inquiry meditation on EEG responses varied according to the type of image viewed: the experienced monks exhibited weaker amplitudes when viewing an image of the self (normal: *M* = 1.22 μV, *SD* = 1.31 μV; inquiry: *M* = 0.41 μV, *SD* = 1.06 μV; *t*(16) = −2.88, *p* = 0.011; EEG amplitudes in the frontal channel Fz, see the last row and 2nd column of Fig. 2b), while the laymen exhibited weaker amplitudes when viewing an image of Buddha (normal: *M* = −1.63 μV, *SD* = 1.10 μV; inquiry: *M* = −1.07 μV, *SD* = 0.81 μV; *t*(17) = 3.12, *p* = 0.0063; EEG amplitudes in the frontal channel Fz, see the 1st row and 3rd column of Fig. 2a). Additionally, the effect of inquiry meditation on EEG responses appeared earlier (140–220 ms) in the monk group than in the control group (220–400 ms; see Fig. 1, Fig. 2).

b. Double dissociation between the two groups

There was a clear double dissociation between the two groups. Participants in the control group showed a greater change in EEG responses (at 220–400 ms) to viewing the image of Buddha in the inquiry condition than in the normal condition (see the first row in Fig. 2a). In contrast, the experienced monk group showed a greater change in EEG responses (at 140–220 ms) to viewing the image of the self. However, monks also had the largest reduction in neural responses to viewing their own images in the inquiry condition compared to the normal condition (140–220 ms, last rows of Fig. 2b). These differences were still significant when compared across groups.

c. Difference between responses to images of the self and Buddha

**Fig. 3:**
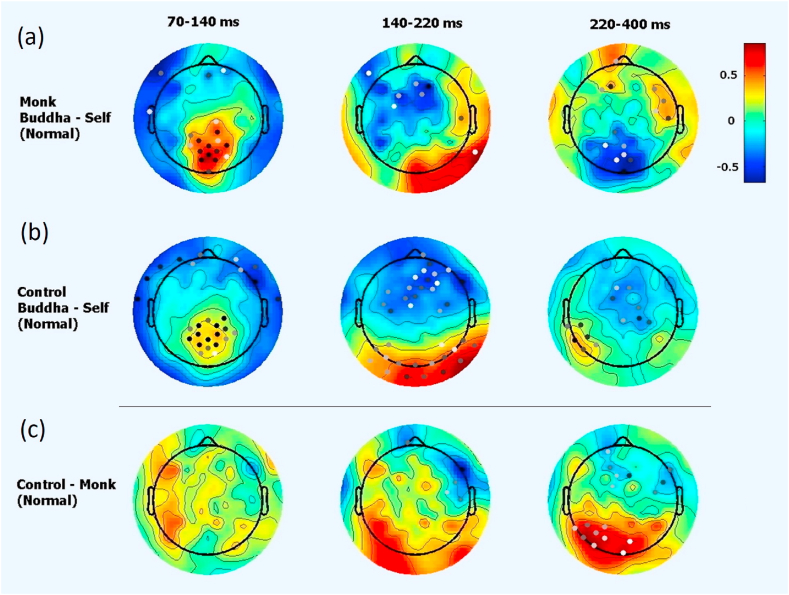
EEG responses under the normal condition when viewing the two types of pictures. There were group differences in EEG responses when viewing pictures of the self vs. Buddha. The power difference is represented by colors. Dots illustrate channels with significant differences (*p* < 0.05), and darker dots indicate smaller p values.

**Fig. 4:**
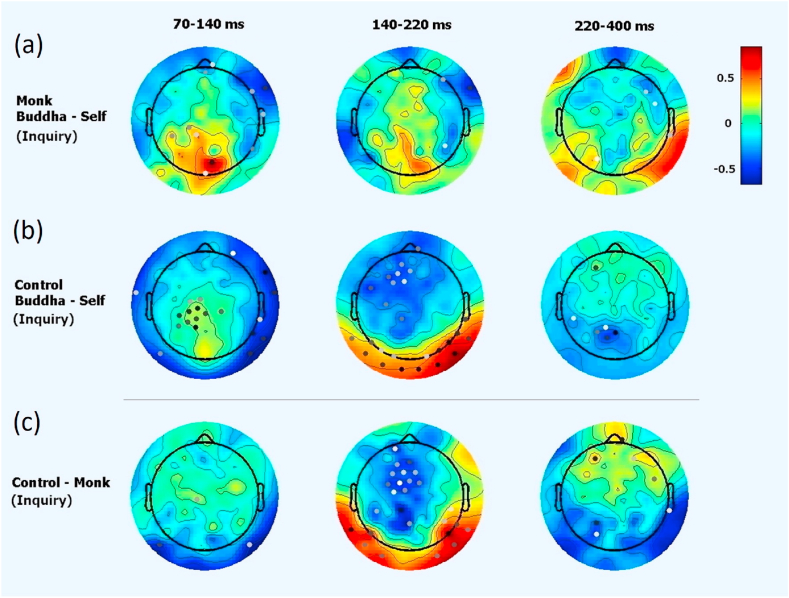
EEG responses under the inquiry condition to viewing two types of pictures. At 140–220 ms, there were significant differences in the response of laymen to pictures of Buddha vs. the self (2nd row); however, no such difference was found among monks (1st row); this difference persisted when further comparing groups (3rd row). The power difference is represented by colors. Dots illustrate channels with significant differences (*p* < 0.05), and darker dots indicate smaller p values.

There was a substantial difference between responses elicited by viewing pictures of the self compared to viewing pictures of Buddha in both groups in the normal condition. The difference was most obvious in the P100/N170 component (see Fig. 3). There were similarities between the groups at this stage. Participants in both groups also exhibited a difference in the P300 component when viewing images of the self and Buddha, although the direction of the effect differed in the two groups. In the experienced monk group, the images of ‘the self’ induced a larger P300 amplitude than images of Buddha (see the first row in Fig. 3) under the normal condition (no meditation), whereas in the layman control group, viewing images of Buddha induced larger P170 and P300 amplitudes than viewing images of the self at the Pz location (during 140–220 ms, see the second row in Fig. 3). Channel Pz is in the medial parietal lobe. These differences mostly persisted in the control group under the inquiry condition (see the second row in Fig. 4). However, these differences mostly disappeared among the monk group, which could imply that the experienced monks might view the concept of the self and Buddha similarly and/or did not differentiate between them during inquiry meditation (see the first row in Fig. 4).

In the right frontotemporal region, viewing the image of Buddha elicited different N170 amplitudes in laymen compared to monks. The difference remained significant when participants viewed images of themselves. These differences were independent of the mediation condition, and they may reflect a general difference in the meditators’ responses to the concepts of Buddha and the self.

d. ERSP and ITC analysis of the Fz channel

The differences between groups and conditions were greater in the frontal area than in other areas. In fact, participants demonstrated a nearly opposite direction of ERP responses in the early stages depending on their group. We further calculated the ERSP values in the normal condition.

**Fig. 5:**
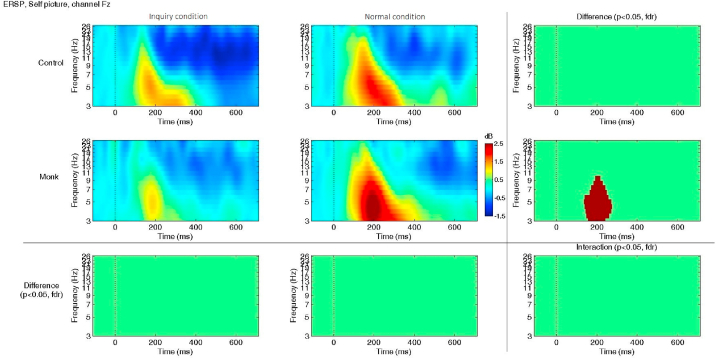
ERSP data from channel Fz exhibit differences between the two conditions when viewing pictures of the self. Significant differences were found at approximately 200 ms among monks (*p* < 0.05, FDR corrected). No significant interaction was found using ANOVA.

ERSP analysis demonstrated a consistent result: only the monk group exhibited greater ERSP values when viewing their own images and a greater reduction in ERSP values under the intuitive inquiry meditation condition (e.g., at 200 ms & 6 Hz, inquiry: *M* = 0.96 dB, normal: *M* = 2.36 dB; *t*(16) = −4.12, *p* < 0.05 FDR corrected; see Fig. 5). The difference was most significant during 180–220 ms and in the theta wave (4–8 Hz).

**Fig. 6:**
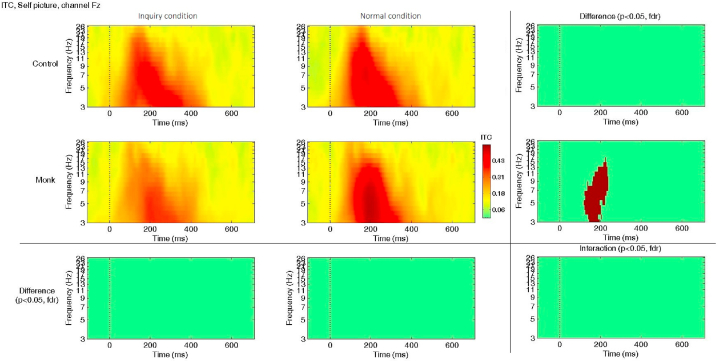
ITC values in channel Fz differed between the two conditions when viewing pictures of the self. Significant differences were found at approximately 200 ms among monks (*p* < 0.05, FDR corrected; 2nd row and 3rd column). No significant interaction was found using ANOVA.

ITC analysis demonstrated that the monks had greater ITC values when viewing their own image; in other words, monks exhibited a greater reduction in ITC values under the intuitive inquiry meditation condition when viewing their own image (e.g., at 200 ms & 6 Hz, inquiry: *M* = 0.35, normal: *M* = 0.51; *t*(16) = −3.77, *p* < 0.05 FDR corrected; see Fig. 6). Both the ERSP and ITC results indicate that monks had greater ERSP changes than laymen under different meditation conditions when viewing images of themselves. The difference was most significant during 180–220 ms and in theta and alpha bands (4–8 Hz, 8–12 Hz).

## 4 Discussion

The results of this ERP study revealed that intuitive inquiry meditation (Chan/Zen meditation, Can-Hua-Tou) could specifically alter a meditator's response to the concept of the self when viewing pictures of oneself or Buddha, which are two important concepts in Buddhism. There was a double dissociation between experienced practitioners (monks) and beginner practitioners (laymen) in this ERP experiment. This implies that training in this form of intuitive inquiry meditation, which involves critical thinking and doubting processes at different levels of practice, may induce different patterns of brain activity when viewing the same two types of images.

The beginners demonstrated a reduced neural response to images of Buddha in the inquiry condition compared to the normal condition**.** There was no such difference in neural responses between the two meditation conditions when participants viewed images of themselves. In contrast, the monks demonstrated a reduced neural response only when viewing their own images in the intuitive inquiry meditation condition compared to the normal condition (no meditation). However, this difference disappeared when these experienced participants viewed an image of Buddha. This result suggests that there are differences in brain activation during intuitive inquiry meditation between experienced meditators and laymen, which could indicate that they may have conceived meditative objects differently. However, future studies with more specific experimental designs are needed to provide more definitive conclusions.

In addition, compared to experienced monks, laymen exhibited differences in the P300 component (220–400 ms), which reflects an attentional process for relatively rare events [24,25]. A previous study found that mindfulness meditation induction can improve accuracy in an attention task, accompanied by a larger P300 amplitude [26]. This shows that during inquiry meditation, laymen focus the doubting process on the concept of Buddha, based on the presented image. This phenomenon might be unique to Buddhist intuitive inquiry mediation which involves an earnest search for enlightenment. In addition to the doubting process during inquiry meditation, other cognitive processes are likely involved in image recognition during meditation. These processes may include attention allocation, perceptual processing, and memory encoding. During the search, practitioners are required to examine the ‘original face’ of everything, including the ultimate authority of the religion, Buddha. The inquiry and doubting process regarding the concept of Buddha is emphasized in a specific group of Buddha texts, the Perfection of Wisdom. For example, the *Diamond Sutra* states that Buddha cannot be conceived by his appearance or his voice, as relying on the outer appearance of Buddha is an incorrect way to perceive and understand the true nature of Buddha.

Among the monks, however, the effect of the doubting process was focused on the concept of the self. This indicates a shift in meditation targets to focus on ‘the self’ (an internal concept), suggesting that they exhibited greater flexibility in self-schema processing. Indeed, in Buddhist practice and theory, the self is the ultimate concern that needs to be addressed and let go of, and ultimate enlightenment can only be reached by releasing the attachment to the concept of the self and attaining anatta (nonself) [27,28]. In the Buddhist tradition, attachment to the concept of the self is the most difficult to break through. In the monk group, the ERP results showed that differences in responses between the meditation and normal conditions occurred in the window of the P100/N170 (during 220–400 ms), an early component for face recognition [29,30]. This ERP result indicates that monks might exhibit altered neural responses to images at the early stage of face recognition, probably due to a top-down process. After long practice with intuitive inquiry meditative, i.e., pondering ‘Who is chanting the name of Buddha?‘, monks might be able to quickly and intuitively alter their mental representation of the self and related information processing. Moreover, perceptual processing, particularly face recognition, appears to play a role in image recognition during meditation. In the experienced monk group, the ERP result showed a difference in the window of the P100/N170—an early component for face recognition—when viewing pictures of themselves in different conditions. This indicates that monks might exhibit altered neural responses to images at the early stage of face recognition, probably due to a top-down process.

According to false tagging theory, monks might have deprioritized the schema of the self through the doubting process induced by the priming question ‘Who is chanting the name of Buddha?‘. Once the doubting process tags the concept of the self as false and/or illusory, the importance of pictures of the self is lessened, and the automatic cognitive processing of the concept of the self is reduced, thus requiring fewer cognitive resources. In Buddhist theory, the self is merely an assembly and aggregation of a variety of components under favorable conditions. Thus, there is no fundamental essence in the concept of self, and it could be subjectively dismantled by careful dialectical analysis [31].

Laymen could be less effective in engaging in this doubting process, as they had a shorter period of practice. Therefore, they could still be focusing on and continuing to attach to external concepts, including the concept of Buddha. The effect of intuitive inquiry meditation was mainly observed on the P300 component (220–400 ms) when viewing an image of Buddha. The P3 component reflects attentional resources for cognitive evaluation. The P300 component in the current study more likely reflects the P3b, as the images were all the same and repeated 60 times in one condition, for a total of 120 times both conditions. The P3 has both P3a and P3b components [32]. The P3a component is referred to as the novel P3, which is attributed to more automatic response processing, probably at frontal lobe generator sites [33,34]. The P3b component is associated with more effortful information processing, which is maximal at the posterior sites [35,36]. In this paper, as the stimuli were frequently repeated, the results reflect the attention allocation process and memory engagement. A previous study found that increased P3b-like amplitudes are associated with successful memory encoding and storage [37]. It is plausible that the evaluation of an image of Buddha engages focal attention, which further facilitates the context maintenance and subsequent memory operations on the concept of Buddha [38,39]. A reduced P3 amplitude during the intuitive inquiry meditation condition implies that laymen allocated fewer cognitive resources to encode the image of Buddha.

In contrast, monks showed little difference in responses to viewing images of Buddha between the inquiry condition and the normal condition. It is conceivable that the experience of monks in engaging in the doubting process might have already led them to reduce cognitive processing of external stimuli, including the image of Buddha.

Some scholars have argued that Buddhism as a religion is unique in terms of belief [40]. While most other religions depend heavily (if not solely) on belief, Buddhist practices urge practitioners to not blindly believe and become attached to any concept, instead encouraging feelings of doubt and inquiry into every concept, even the concept of Buddha. Practically, this doubting practice has two purposes in intuitive inquiry meditation: first, doubt can be inspired by any concept or term. As described by the Buddhist doctrine, external phenomena and internal reflections are not independent and thus are eventually subject to change and deteriorate. Second, the doubting process itself can be an object of meditation, providing additional metacognition to monitor this ongoing doubting process [41].

In the experienced monk group, the object of intuitive inquiry meditation seems to have shifted to the more fundamental concept of ‘the self’, a form of metacognition that involves deep reflexive ability. The ERP results showed the greatest reduction in amplitudes in the P100/N170 window when monks processed images of themselves. Face recognition can elicit the N170 component in the temporal-occipital region, accompanied by a more frontal positive component, referred to as the P170 or vertex positive potential (VPP) [42,43]. The P170, similar to the N170, is mostly related to perceptual processing during face recognition and attention reallocation. However, the P170 has different characteristics and occurs in a more frontal region. For example, the frontal P170 component is not observed in children aged 7–8 years, although they do exhibit the N170 component elicited by face recognition. The P170 only starts to appear in adolescents and becomes stable in adults [44]. A recent ERP study on insight found that the frontal P170 amplitude increases when individuals face cognitive challenges, such as riddles [45]. A subsequent study also showed that the P170 and P2 may reflect an unconscious metacognitive monitoring process. According to the dual-process framework of metacognition, metacognition has two processes: metacognitive feelings, which are mainly based on nonanalytic processes, and metacognitive judgments, which are based on analytic processes [46]. Additional ERP studies on insight have also suggested that the P170 component may be automatic and occur without much conscious processing at an early stage of information processing [[47], [48], [49]].

In the current study, monks exhibited greater frontal P170 (140–220 ms) amplitudes when viewing images of themselves in the normal condition than in the inquiry condition. These monks have practiced intuitive inquiry meditation for many years; thus, it is plausible that even in the normal condition, they engaged in doubting process when viewing images of themselves, while in the inquiry condition, they might have already reached a state of detachment from the self. Intuitive inquiry meditation as a practice has the aim of directly contemplating and analyzing the issue of ‘the self’ to attain a state of ‘nonself’, in which self-identity is no more than an assembly of dependent conditions [50,51].

After long-term practice, monks demonstrated greater flexibility between the different conditions. According to the ERSP results, monks showed the greatest reduction in ERSP values in the theta band when viewing images of themselves in the inquiry condition compared to viewing images of themselves in the normal condition. Notably, theta band activity is frequently observed in the performance of various memory and cognitive control tasks [52]. A reduction in theta band activity during the inquiry condition may indicate a lower demand for cognitive resources, probably due to the practice of inquiry meditation. By contemplating the impermanence of the self, monks can quickly let go of their attachment to the self. Theta band activity has been frequently observed during cognitive control performance and cross-frequency coupling, especially in deeper brain regions, such as the hippocampus. One previous EEG study found that theta phase-gamma amplitude coupling plays an important role in cognitive processing and reflects information processing and signaling across distant brain regions [53].

At the same time, the ITC results showed the greatest reduction in ITC values in the inquiry condition compared to the normal condition when viewing images of the self. ITC represents the trial-to-trial coherence of brain processing of similar stimuli, reflecting the stability of the neural response [54,55]. A higher ITC value implies a more stable response, while a lower ITC value indicates that less attention was given to specific stimuli during processing, indicating neuroplasticity [56]. These differences were not observed in beginners. The ERSP and ITC results indicate that monks exhibited greater changes in brain activity between tasks than laymen. In other words, monks flexibly recruited more cognitive resources in a specific task while greatly reducing the resources recruited during intuitive inquiry meditation. This finding aligns with the Buddhist emphasis on the wisdom of nonattachment and letting go since all phenomena are impermanent, including oneself. Thus, it is imperative to be dynamic and flexible when dealing with ongoing events. While the doubting process is a key component of intuitive inquiry meditation, other cognitive processes, such as attention allocation, perceptual processing, and memory encoding, also play important roles in image recognition during meditation. Understanding these processes can provide a more comprehensive view of the cognitive changes induced by meditation and contribute to our understanding of the neural markers for long-term intuitive inquiry meditation.

Further source localization analyses of cortical sources provided additional evidence for the effects observed in the frontal region related to the doubt process. This source analysis of prefrontal activity demonstrated that the prefrontal region plays a key role in meditative practices and further underscores the potential neural changes induced by long-term meditation practice. The source analysis revealed a significant interaction between condition and group in the frontal region, aligning with the Fz channel result. Due to greater cognitive flexibility, monks exhibited a similar brain response to viewing an image of Buddha and an image of themselves. In contrast, laymen differed in the brain responses to viewing an image of Buddha and an image of themselves. This difference between groups remained significant. This illustrates that experienced monks can reach a state of equanimity toward the two stimuli [57] and evenness of mind even under attention-attracting concepts, such as Buddha and the self. Thus, even in the layman control group, the difference between the responses to viewing images of oneself and Buddha was smaller in the inquiry condition than in the normal condition. The difference in responses to viewing images of the self and Buddha was also obvious among monks in the normal condition. This further reflects the ability of monks to dynamically control the cognitive processing of images, as suggested by the Buddhist theory of the wisdom of nonattachment.

As Anattalakkhana Sutta (in Pali) repeatedly emphasizes, all phenomena, whether external or internal, in the body or mind, have characteristics of impermanence and nonessence (self) [58,59]. Nothing is truly dependable or reliable, nor can anything be claimed to be ‘mine’ all the time, nor is there a real and unchanging ‘me’. Thus, suffering cannot be prevented due to this impermanence and lack of the self. By reflecting on this wisdom, practitioners can become more aware of the true nature of Buddha and the self, although the sophistication and emphasis are different between beginners and experienced monks, as demonstrated by our ERP results.

During intuitive inquiry meditation, monks demonstrated the ability to reduce the difference in brain activity in response to the self and other concepts, including Buddha. In contrast, laymen exhibited a significant difference in brain activity when viewing images of the self and Buddha during the inquiry condition. This result indicates that beginners were unable to eliminate the difference between the two concepts, the self and Buddha, to the extent demonstrated by experienced meditators.

a. Limitations and future studies

Several limitations are worth noting in the current study. First, intuitive inquiry meditation is usually a pondering and doubting process that occurs in the mind. This can be better measured over a longer period. This ERP experiment might be too short to capture the full changes in brain activity induced by inquiry meditation. Moreover, the doubting process induced by viewing images during meditation can be superimposed on other cognitive processes, such as attention allocation and image recognition. This complexity imposes a limitation on the interpretation of our results, as it is difficult to isolate this cognitive process from others and conclusively determine the effects of Buddhist Chan practice on the doubting process alone. Future studies could aim to further disentangle these intertwined cognitive processes. Given the complexity of the doubting process, more participants may be needed in future studies. A longer experimental paradigm or functional magnetic resonance imaging may better illustrate the neural correlates of intuitive inquiry meditation, especially those in the subcortical regions. Another limitation is that the beginner participants performed their experiments in an EEG laboratory, while all the monks completed the experiment in a temple. The experiments were limited by the time constraints of the inquiry meditation retreat, which lasted 7 days; thus, it was not feasible to collect all the data within that short period.

## Author contribution statement

Junling Gao; Hang Kin Leung: Conceived and designed the experiments; Performed the experiments; Analyzed and interpreted the data; Wrote the paper. Bonnie Wai Yan Wu; Chunqi Chang; Jenny Hung: Contributed reagents, materials, analysis tools or data. Hin Hung Sik: Conceived and designed the experiments; Contributed reagents, materials, analysis tools or data; Wrote the paper.

## Data availability statement

Data will be made available on request.

## Compliance with ethical standards

This study received ethical approval from the Human Research Ethics Committee (HREC) at the University of Hong Kong, the reference number is EA1606043. Informed consent was obtained from all participants included in the study.

## Declaration of competing interest

The authors declare that they have no known competing financial interests or personal relationships that could have appeared to influence the work reported in this paper.
