# Supplementary material for: Long-term practice of intuitive inquiry meditation modulates EEG dynamics during self-schema processing
Source: Heliyon. 2023 Sep 12;9(9):e20075. doi: 10.1016/j.heliyon.2023.e20075 (PMC10559825; doi:10.1016/j.heliyon.2023.e20075)
Supplement: Multimedia component 1 [file mmc1.docx]

**Supplementary**


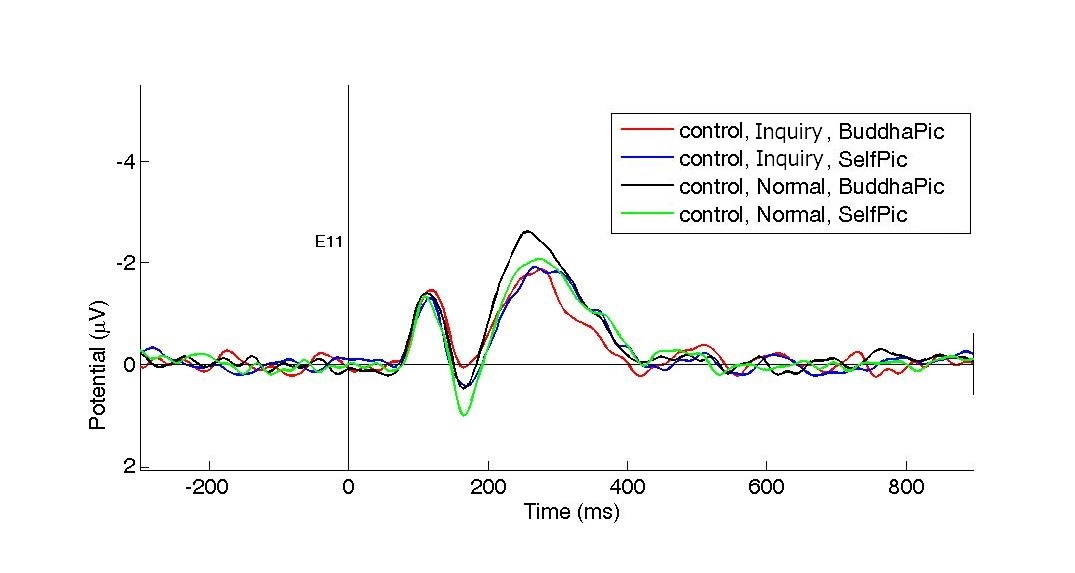


**sFigure 1.** Layman control group’s ERP of two meditation conditions (inquiry vs. normal) for the two picture types (Buddha vs. self) at channel Fz.


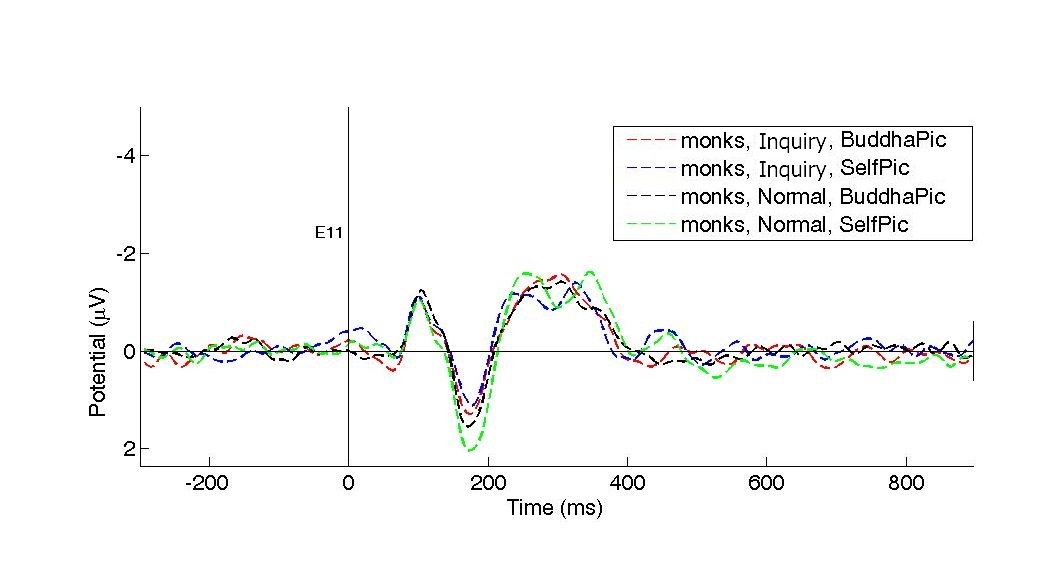


**sFigure 2.** Monks’ ERP of two meditation conditions (inquiry vs. normal) for the two picture types (Buddha vs. self) at channel Fz.


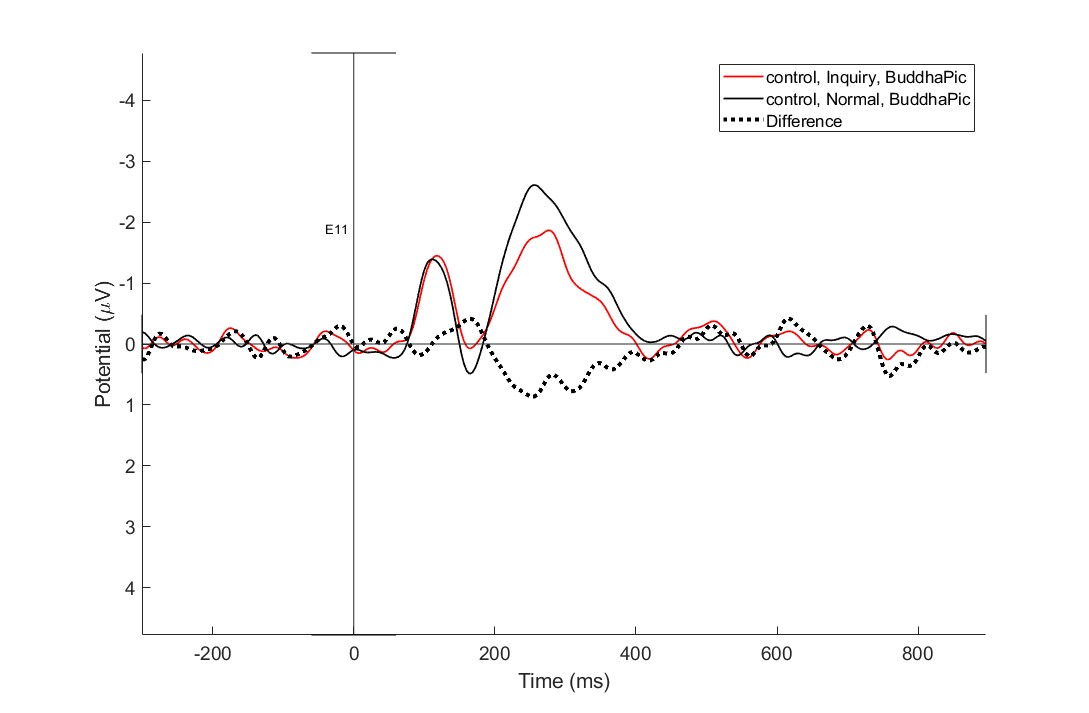


**sFigure 3.** Control’s ERP wave difference between inquiry and normal conditions while viewing the Buddha picture. E11 is the channel Fz.


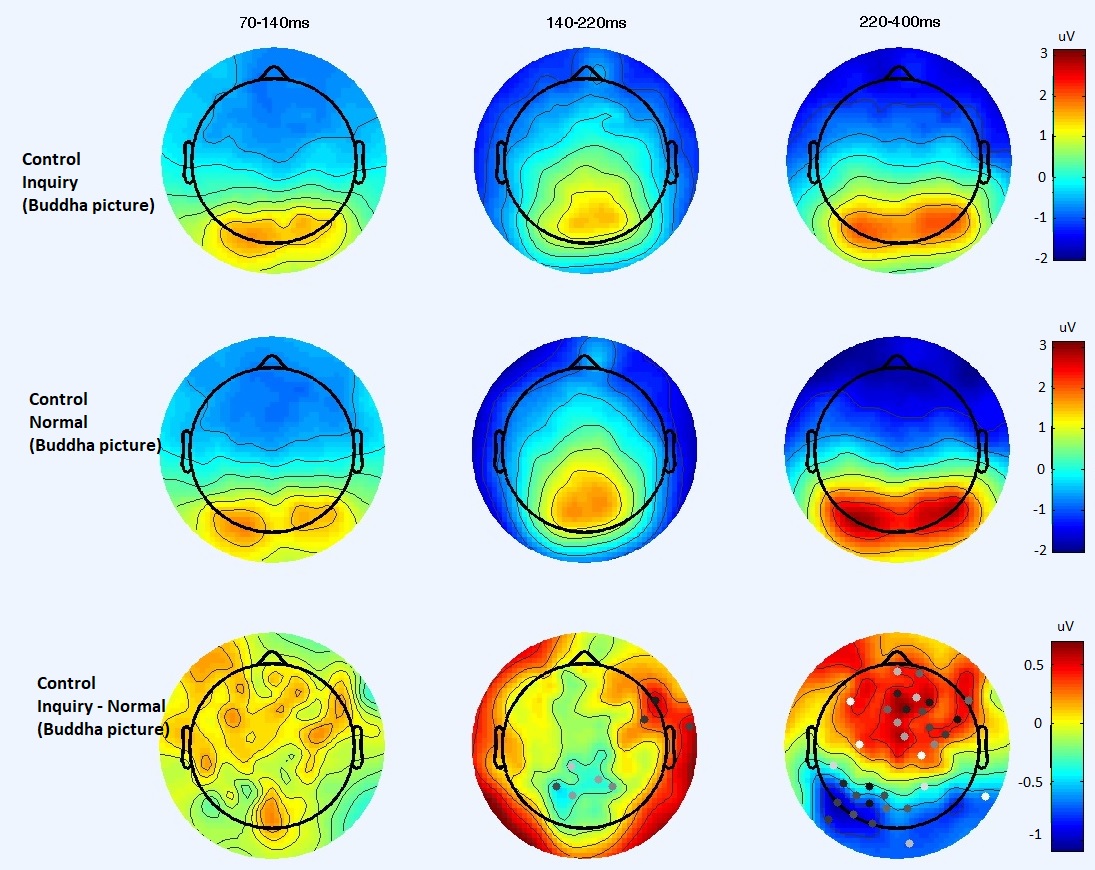


**sFigure 4.** Control’s ERP topographic map of inquiry and normal conditions while viewing the Buddha picture. Dots illustrate channels with significant differences (p < 0.05), and darker dots indicate smaller p-values.


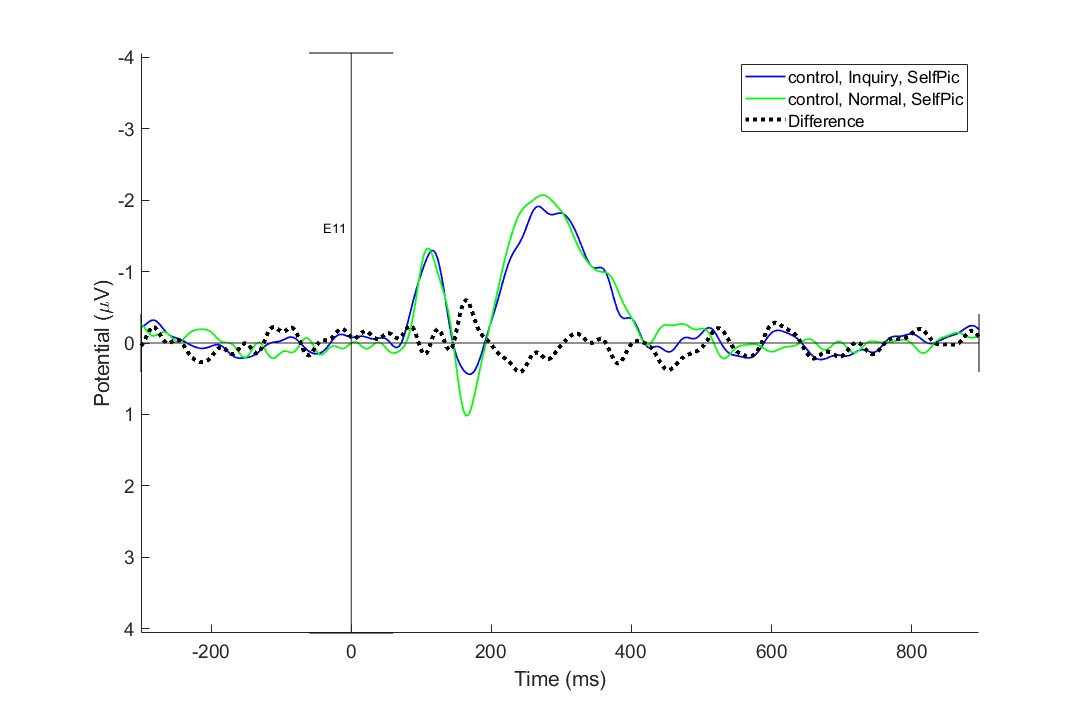


**sFigure 5.** Control’s ERP wave difference between inquiry and normal conditions while viewing picture of self. E11 is the channel Fz.


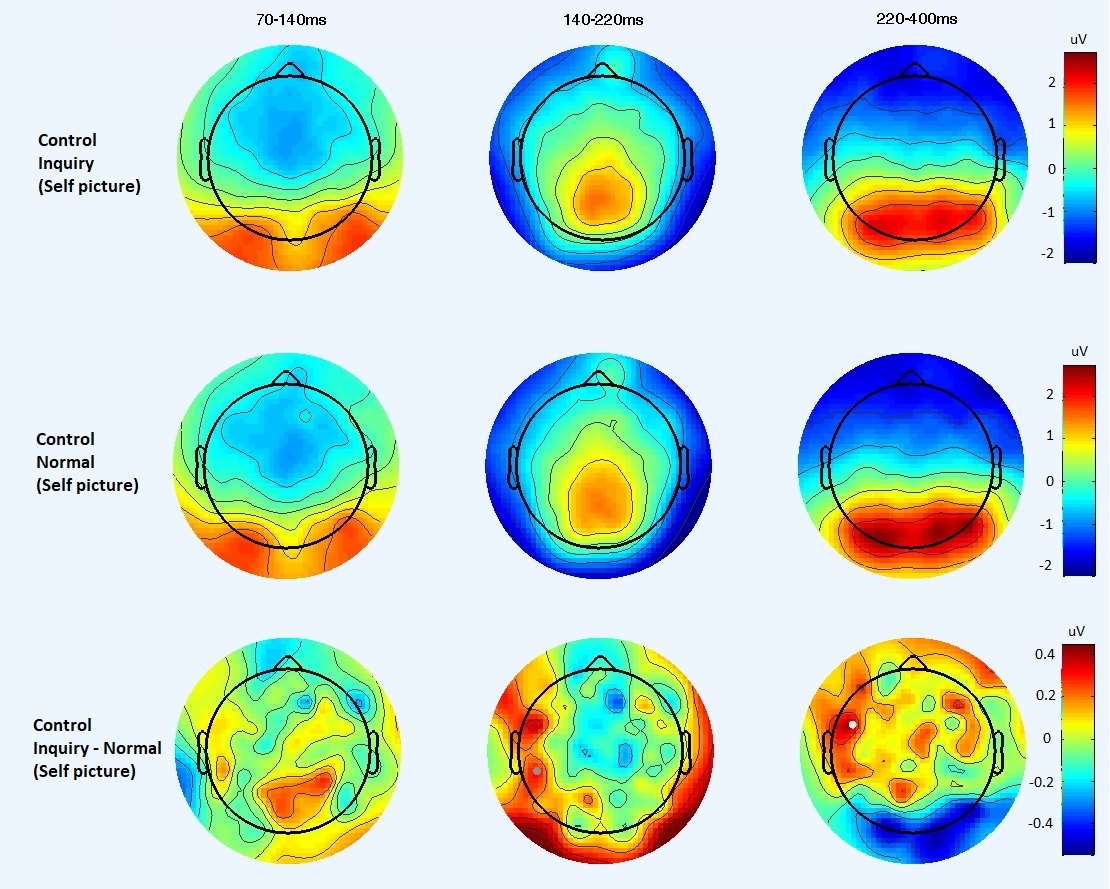


**sFigure 6.** Control’s ERP topographic map of inquiry and normal conditions while viewing picture of self. Dots illustrate channels with significant differences (p < 0.05), and darker dots indicate smaller p-values.


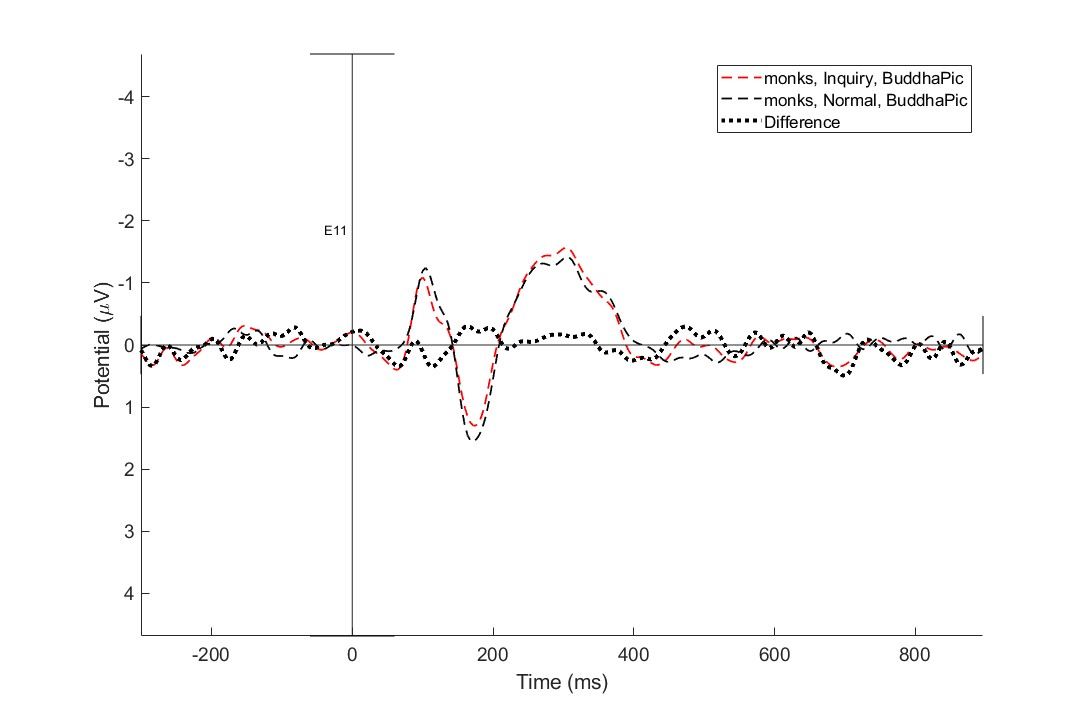


**sFigure 7.** Monk’s ERP wave difference between inquiry and normal conditions while viewing the Buddha picture. E11 is the channel Fz.


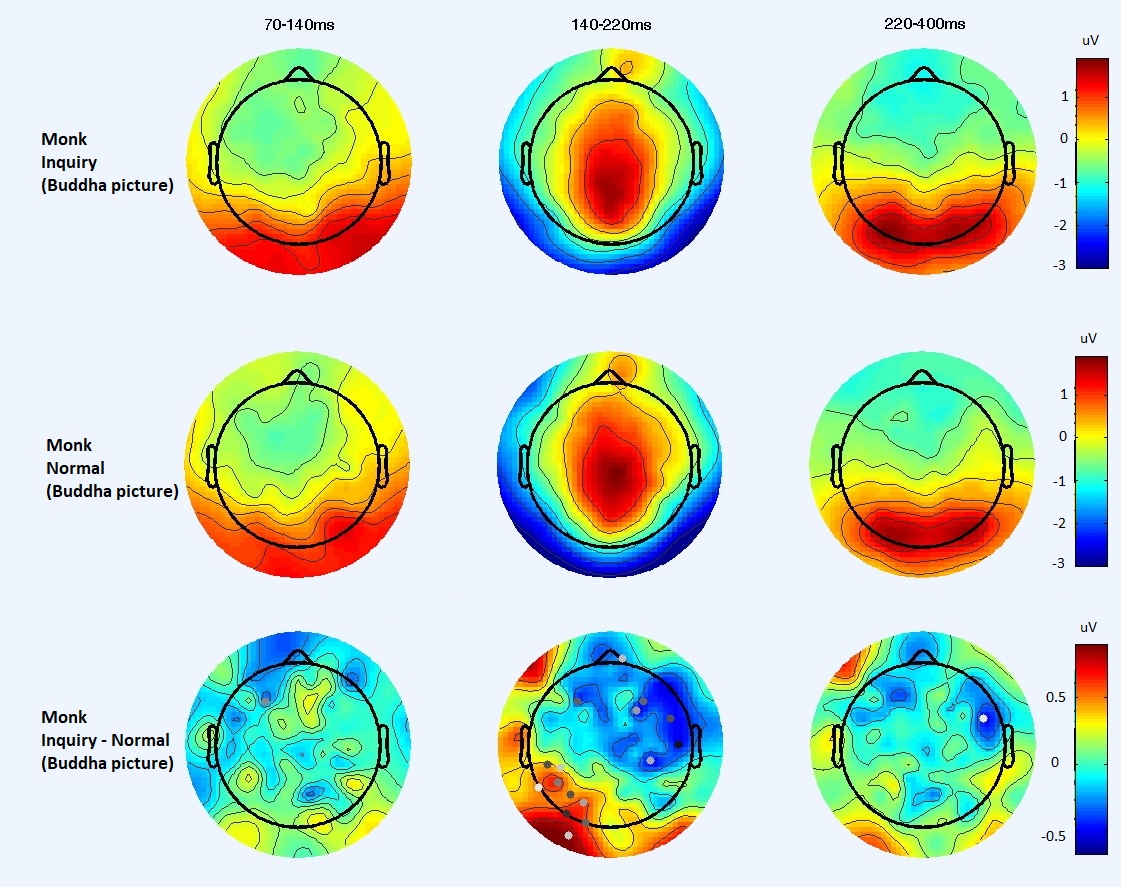


**sFigure 8.** Monk’s ERP topographic map of inquiry and normal conditions while viewing picture of Buddha. Dots illustrate channels with significant differences (p < 0.05), and darker dots indicate smaller p-values.


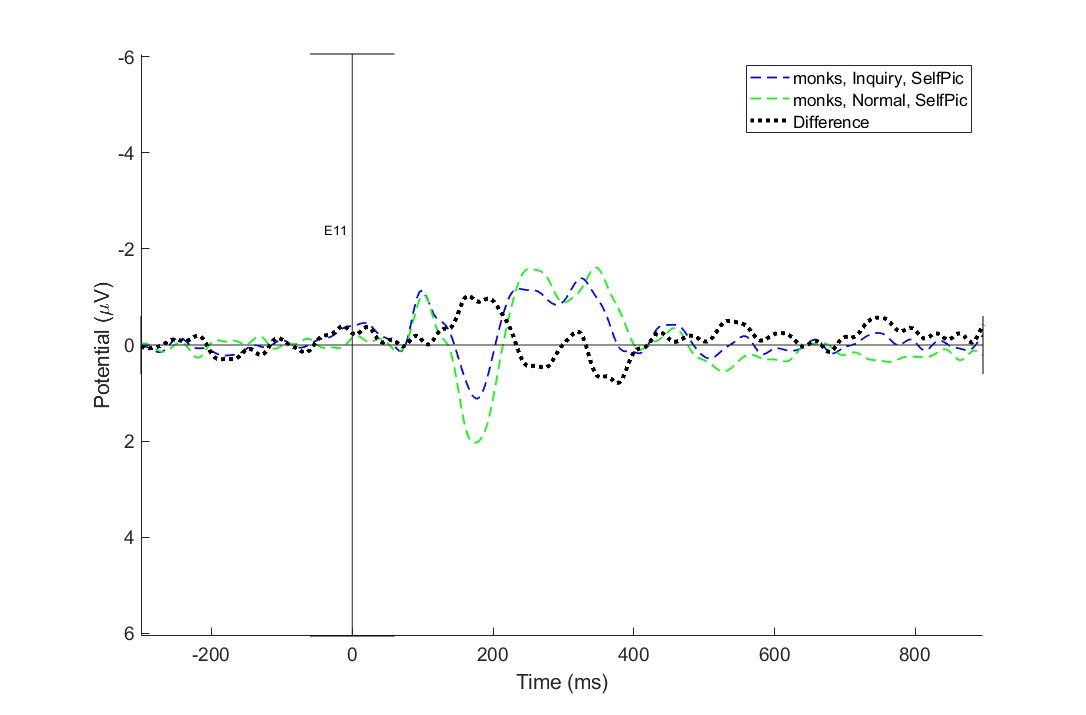


**sFigure 9.** Monk’s ERP wave difference between inquiry and normal conditions while viewing their picture of self. E11 is the channel Fz.


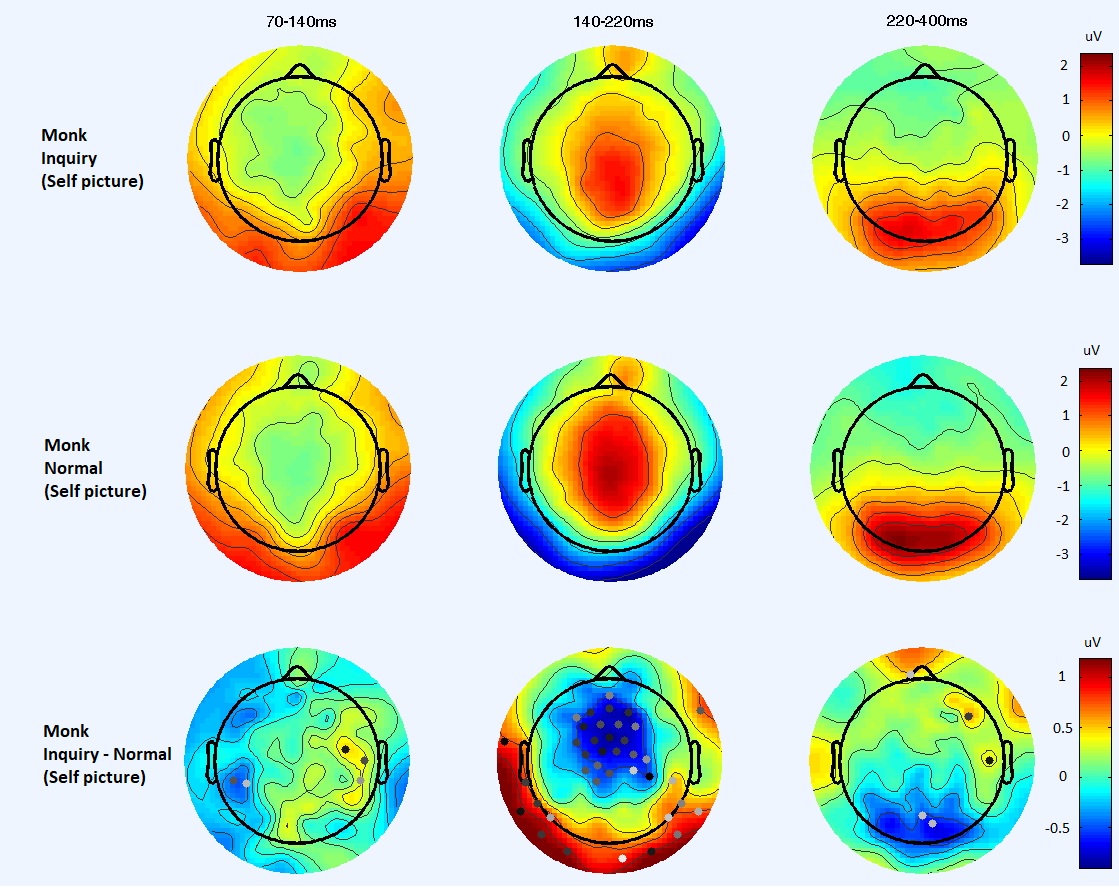


**sFigure 10.** Monk’s ERP topographic map of inquiry and normal conditions while viewing picture of self. Dots illustrate channels with significant differences (p < 0.05), and darker dots indicate smaller p-values.


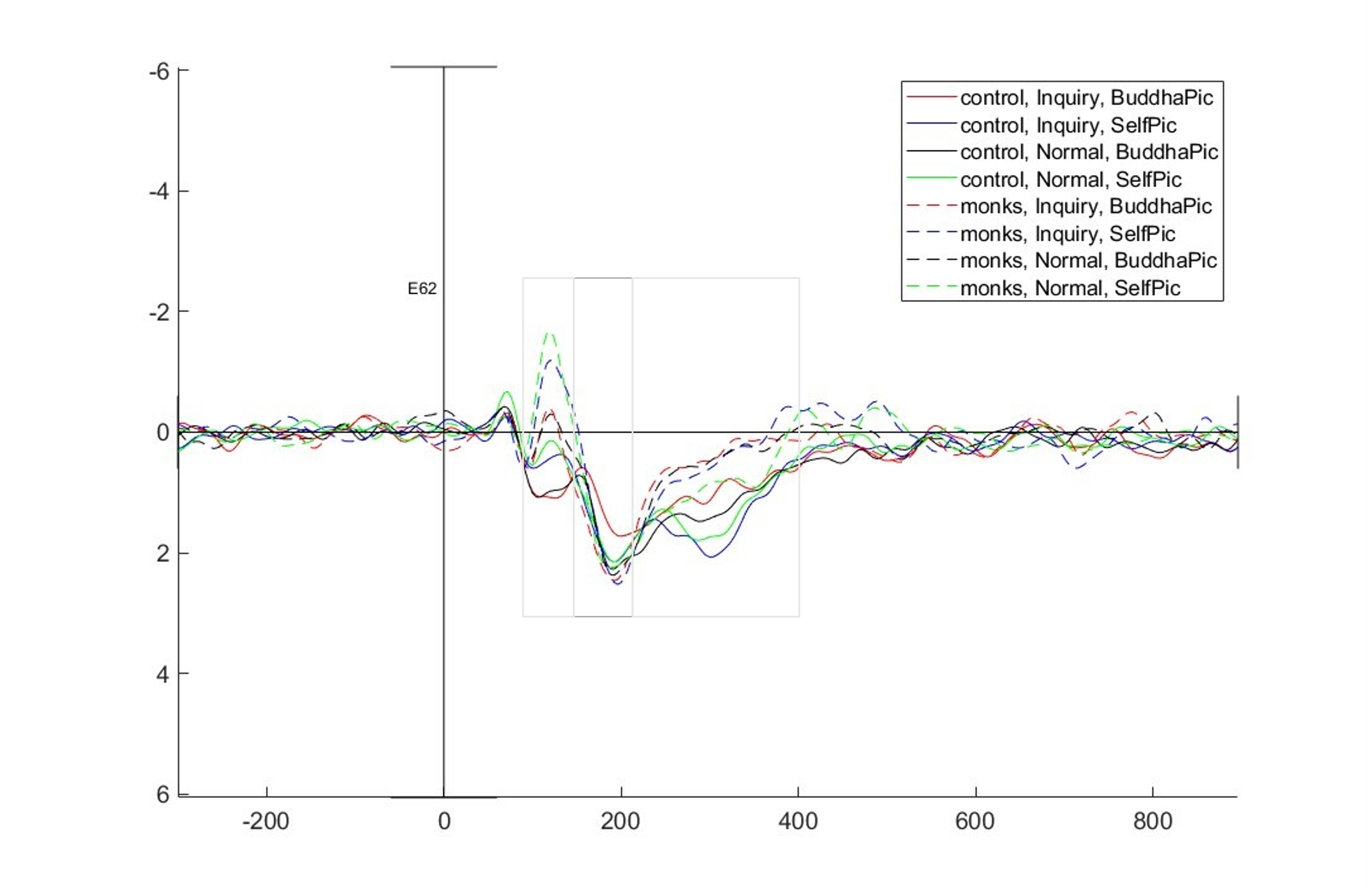


**sFigure 11.** ERP waveforms of two meditation conditions (inquiry vs. normal) for the two picture types (Buddha vs. self) in the two groups (experienced monks vs. layman control) at channel Pz. Thicker solid lines represent the control group.


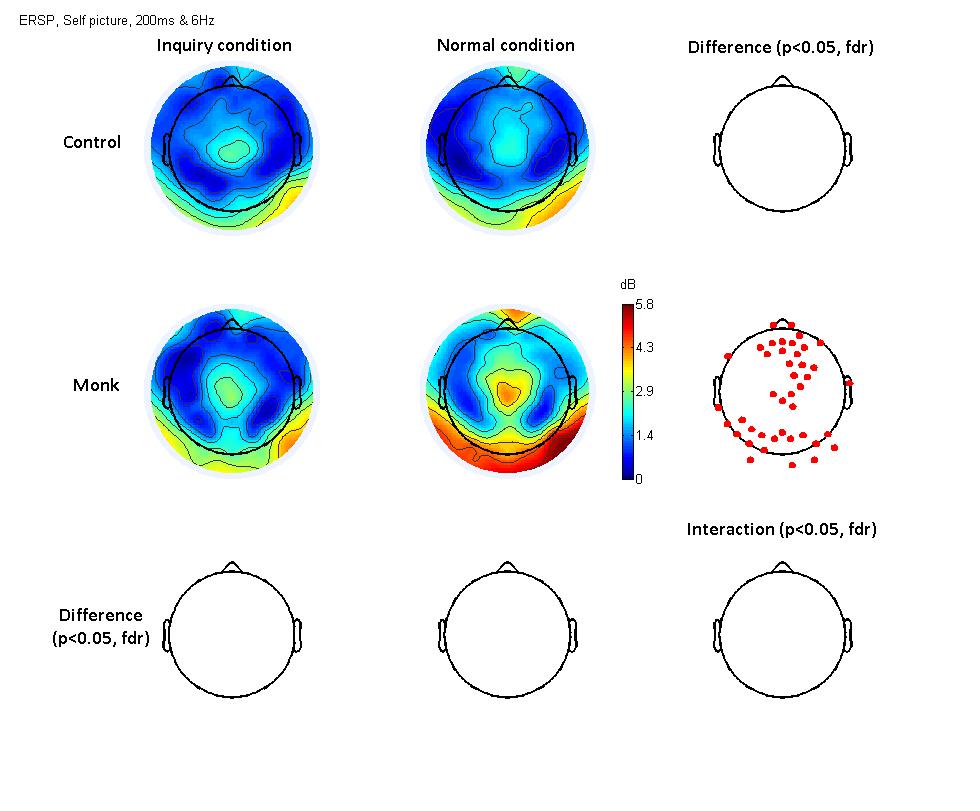


**sFigure 12.** ERSP topography at 200 ms & 6 Hz while viewing image of self. Dots illustrate channels with significant differences (p < 0.05, fdr corrected).


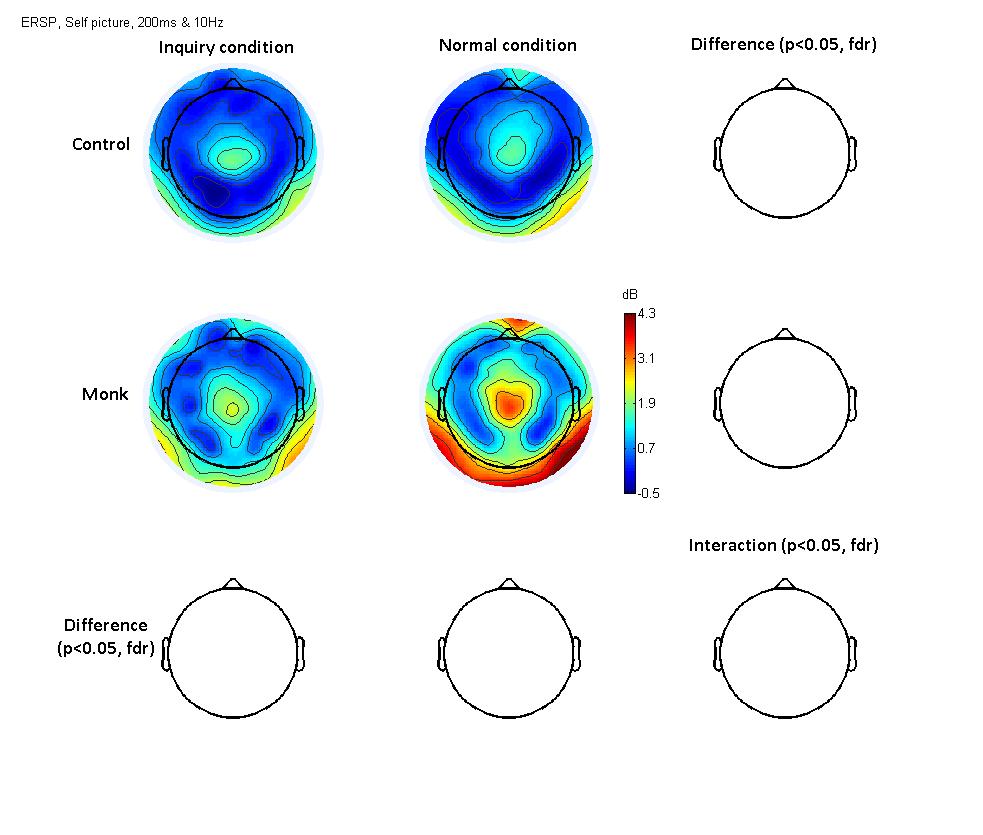


**sFigure 13.** ERSP topography at 200 ms & 10 Hz while viewing image of self. No significant difference was found.


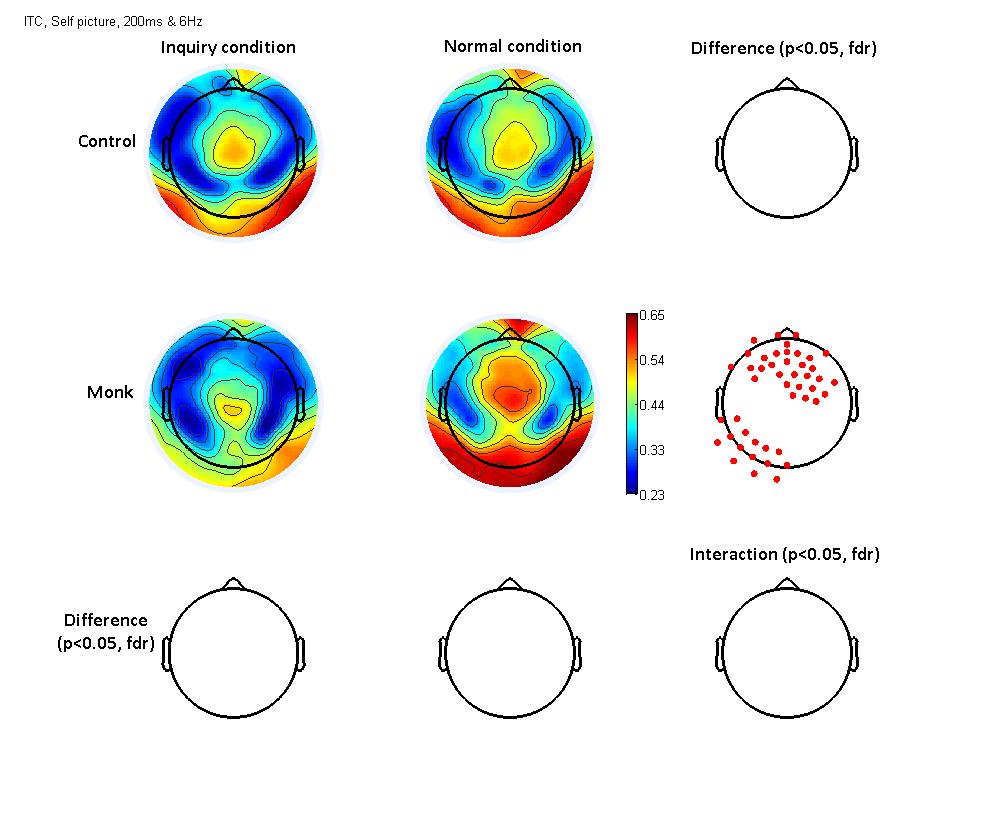


**sFigure 14.** ITC topography at 200 ms & 6 Hz while viewing image of self. Dots illustrate channels with significant differences (p < 0.05, fdr corrected).


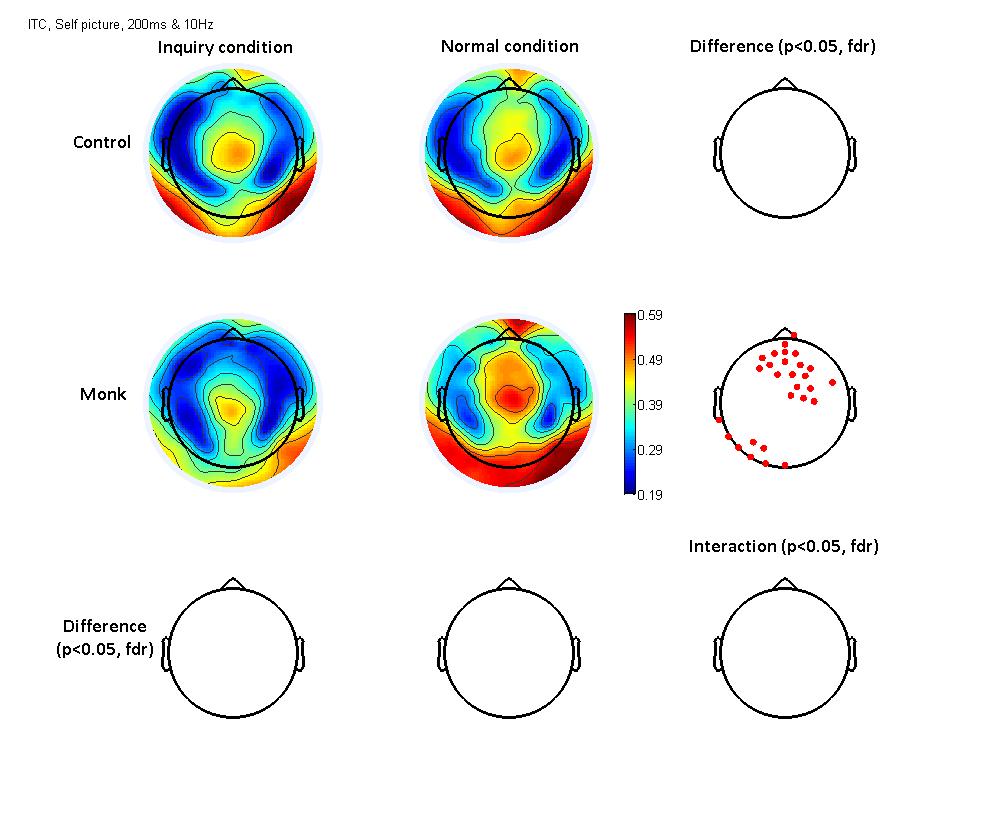


**sFigure 15.** ITC topography at 200 ms & 10 Hz while viewing image of self. Dots illustrate channels with significant differences (p < 0.05, fdr corrected).


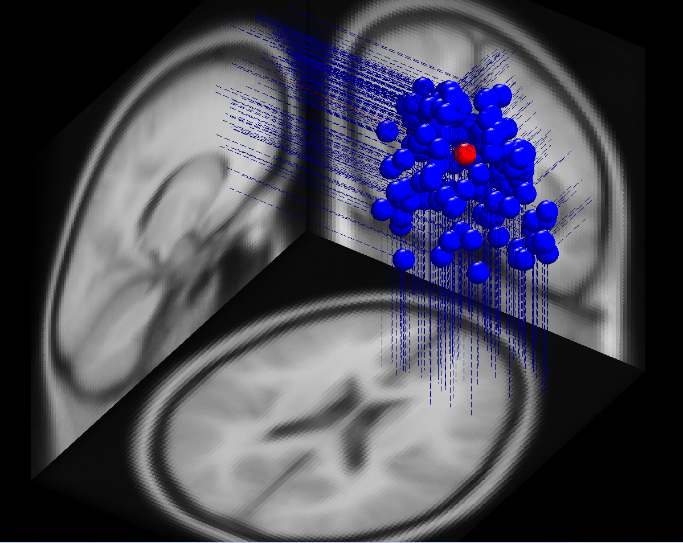
**
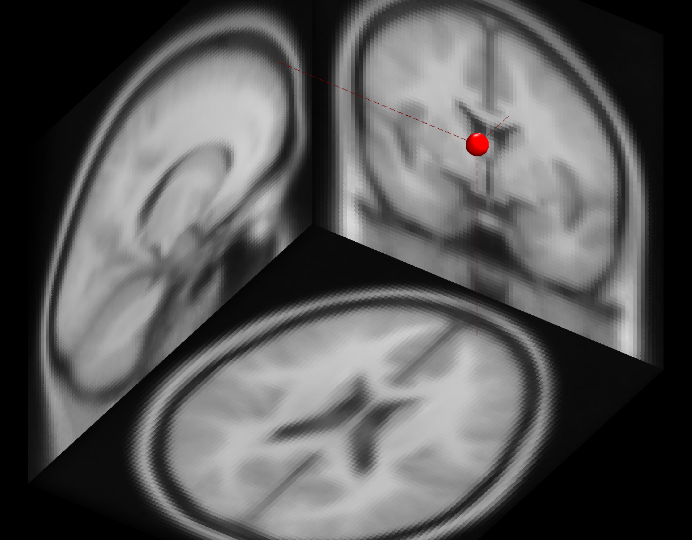
**

**sFigure 16.** The frontal cluster of dipoles obtained by dipole fitting and the *k*-means clustering algorithm. Red dipole is the cluster centroid. For cortical localization, we used the DIPFIT plugin of EEGLAB and the k-means algorithm to form clusters, and clustering criteria were based on dipole locations of independent components (ICs), with a specific focus on the frontal area. The clustering process was initiated with *k*=6 and ended at *k*=10. Then, we analysed how the frontal area was formed over different k values and examined whether the identified frontal cluster would be consistently generated over different *k* values. The frontal cluster was consistently generated, with *k*=8 producing a significant interaction (*p* <.05, fdr corrected) result that aligned with the main result. No other fdr-corrected significance was found in ERP and ERSP data.


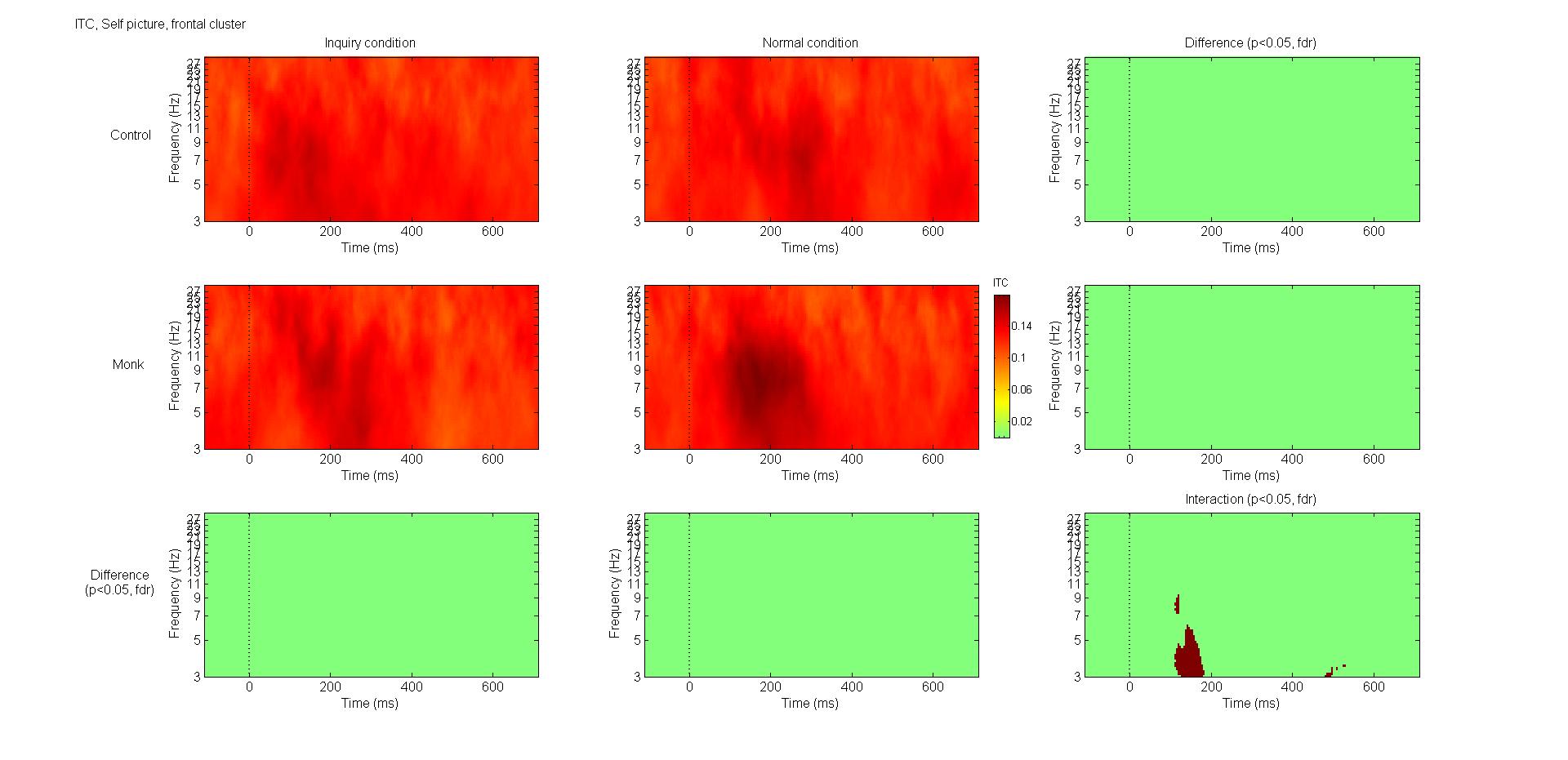


**sFigure 17.** ITC analysis of the frontal cluster shows significant interaction between the group factor and the condition factor, while viewing picture of self. This cluster was generated with *k*=8 in the *k*-means algorithm. Significant interaction can be clearly identified at 180 ms & 3-6 Hz (p < 0.05, fdr corrected).
